# Lead exposure in relation to gut homeostasis, microbiota, and metabolites

**DOI:** 10.1128/aem.00372-25

**Published:** 2025-07-03

**Authors:** Yixuan Tao, Dongling Liu, Qianhan Shi, Qinghua Sun, Cuiqing Liu, Xiang Zeng

**Affiliations:** 1 School of Public Health, Zhejiang Chinese Medical University 70571, Hangzhou, Zhejiang, China; 2 School of Basic Medical Science, Zhejiang Chinese Medical University 70571, Hangzhou, Zhejiang, China; 3 Zhejiang International Science and Technology Cooperation Base of Air Pollution and Health, Zhejiang Chinese Medical University 70571, Hangzhou, Zhejiang, China; Colorado School of Mines, Golden, Colorado, USA

**Keywords:** lead (Pb), toxicity, microbiota, metabolize, gut health

## Abstract

Lead (Pb) is a hazardous heavy metal with no known safe threshold for exposure or consumption, posing significant risks to human health. Pb exposure can cause multiple system damage, depending on exposure levels, duration, and its high bioavailability and bioaccumulative potential. Gastrointestinal tract serves as a primary site for Pb absorption, making it particularly vulnerable to Pb-induced damage, including disruption of gut microbiota composition and metabolic function. This study briefly summarizes the detrimental effects of Pb gut homeostasis, microbial ecology, and host metabolism, which, in turn, further contribute to systemic toxicity. Notably, Pb exposure compromises intestinal barrier integrity, increasing gut permeability and facilitating the translocation of harmful biomolecules into systemic circulation, thereby exacerbating organ dysfunction. Importantly, we underscore that dietary and nutritional interventions such as fiber, probiotic, and vitamin C supplementation is a practicable and effective strategy for mitigating or preventing Pb toxicity.

## INTRODUCTION

Lead (Pb) and its compounds are pervasive environmental pollutants extensively utilized in industrial and consumer products, including batteries, plastics, glass, paints, alloys, pesticides, coatings, ammunition, fuel additives, skin care products, and electronic waste (e-waste) (1). Despite global efforts to mitigate Pb contamination such as phasing out leaded gasoline and regulating lead-based paints, anthropogenic activities continue to elevate Pb levels in the biosphere far beyond natural background concentrations (2). Alarmingly, approximately 800 million children worldwide (one-third of all children) have blood lead levels (BLLs) exceeding the WHO recommended action threshold (5 µg/dL), with around 90% of these cases occurring in low- and middle-income countries (LMICs) (3). Regions such as South Asia, sub-Saharan Africa, and Latin America are disproportionately affected, largely due to informal Pb-related industries and inadequate regulatory measures. For instance, Nigeria’s artisanal gold mining has led to soil Pb concentrations reaching up to 185,000 mg/kg that exceeds the WHO limit of 400 mg/kg by 462.5 times, and outbreak of fatal childhood Pb poisoning has killed approximately 400 children < 5 years old and over 2,000 children left with permanent disabilities (4–6). Similarly, informal Pb-acid battery recycling in Senegal has resulted in median BLLs of 129 µg/dL among nearby children—25 times the WHO safety limit (7). The economic burden is equally staggering, with Pb exposure in LMICs causing 1.2%–2.3% GDP losses annually, primarily due to cognitive impairments and reduced productivity (8). These examples underscore the urgent need for global intervention strategies.

Exposure to Pb not only has no benefits for the human body but can also cause irreversible damage to the body (9–11). Pb enters the human body through ingestion, inhalation, and skin absorption, subsequently traversing biological barriers such as the alveolar blood-air barrier and intestinal blood-gut barrier, and ultimately accumulating in vital organs and tissues in the human body (12). The half-life of Pb varies significantly across tissues, ranging from 30 days in blood to 30 years in bone (13). In adults, around 94% of Pb is deposited in bones and teeth, whereas children retain an average of 73%, reflecting their heightened susceptibility due to ongoing skeletal development. The remaining fraction distributes into blood (99% bound to erythrocytes, 1% in plasma) and soft tissues, with the potential to breach the blood-brain barrier (BBB), inducing neurotoxicity and neurodegenerative disorders (14). The gastrointestinal (GI) tract serves as the primary entry point for dietary and environmental Pb, with infants and children absorbing 42%–50% of ingested Pb, compared to only 5%–10% in adults (15). Consequently, pediatric populations face 4-5 times greater exposure risks, with 80%–90% of total Pb uptake attributed to contaminated food and water (16). Major dietary sources of Pb include cereals (24.1%), beverages (14.3%), vegetables (10.7%), dairy products (9.7%), fruits (9.3%), meat and fish (3.4%) (17). Global disparities in Pb intake are stark: daily consumption averages 37.1 µg (Germany), 35.1 µg (China), 14.7 µg (Spain), and 3.5 µg (USA)—exceeding the FDA’s provisional tolerable intake (12.5 µg/day) in multiple regions (18–20). Correspondingly, mean pediatric BLLs reflect these trends: 3.71 µg/dL (China), 1.10 µg/dL (Germany), and 0.84 µg/dL (USA) (18–20). Pb toxicity is mediated through multiple mechanisms as follows. First, the large ionic radius and high electronegativity of Pb enable binding to proteins, nucleic acids, and lipids, impairing cellular function (12). Additionally, Pb induces reactive oxygen species (ROS; e.g., O₂⁻, H₂O₂, NO), triggering DNA damage, lipid peroxidation, and aberrant protein modifications (21). Moreover, Pb competitively inhibits absorption of essential minerals (e.g., Ca²^+^, Fe²^+^), disrupting metabolic homeostasis (22). Furthermore, as an endocrine-disrupting chemical (EDC), Pb contributes to neurodevelopmental deficits, hypertension, nephrotoxicity, and reproductive harm (1). Notably, Pb accumulates and magnifies across food chains, ultimately reaching harmful levels in humans (23). Finally, chronic Pb exposure is associated with the formation and development of benign and malignant tumors (24, 25).

The human gut harbors approximately 2,000 bacterial species with a collective population of 10¹⁴ microbial genomes, exceeding the human genome by 100-fold (26, 27). Gut microbiota can be considered a functional metabolic “organ” which maintains intestinal barrier integrity, immune regulation, and metabolic homeostasis (28, 29). Dominant phyla include *Firmicutes*, *Bacteroidetes*, *Proteobacteria*, *Actinobacteria*, and *Fusobacteria*, with optimal health linked to diversity, stability, and host symbiosis (30). It is noteworthy that Pb disrupts gut microbiota homeostasis through three primary mechanisms: microbial dysbiosis, gut barrier impairment, and immune modulation. For example, Pb exposure depletes commensal probiotics (*Lactobacillus*, *Bifidobacterium*) while promoting pathogenic overgrowth (31, 32); reactive oxygen species (ROS) triggered by Pb exposure degrade tight junction proteins inducing leaky gut syndrome (33–36); Pb disrupts antimicrobial peptide production and microbial nutrient competition, amplifying systemic inflammatory responses (37, 38).

**Fig 1:**
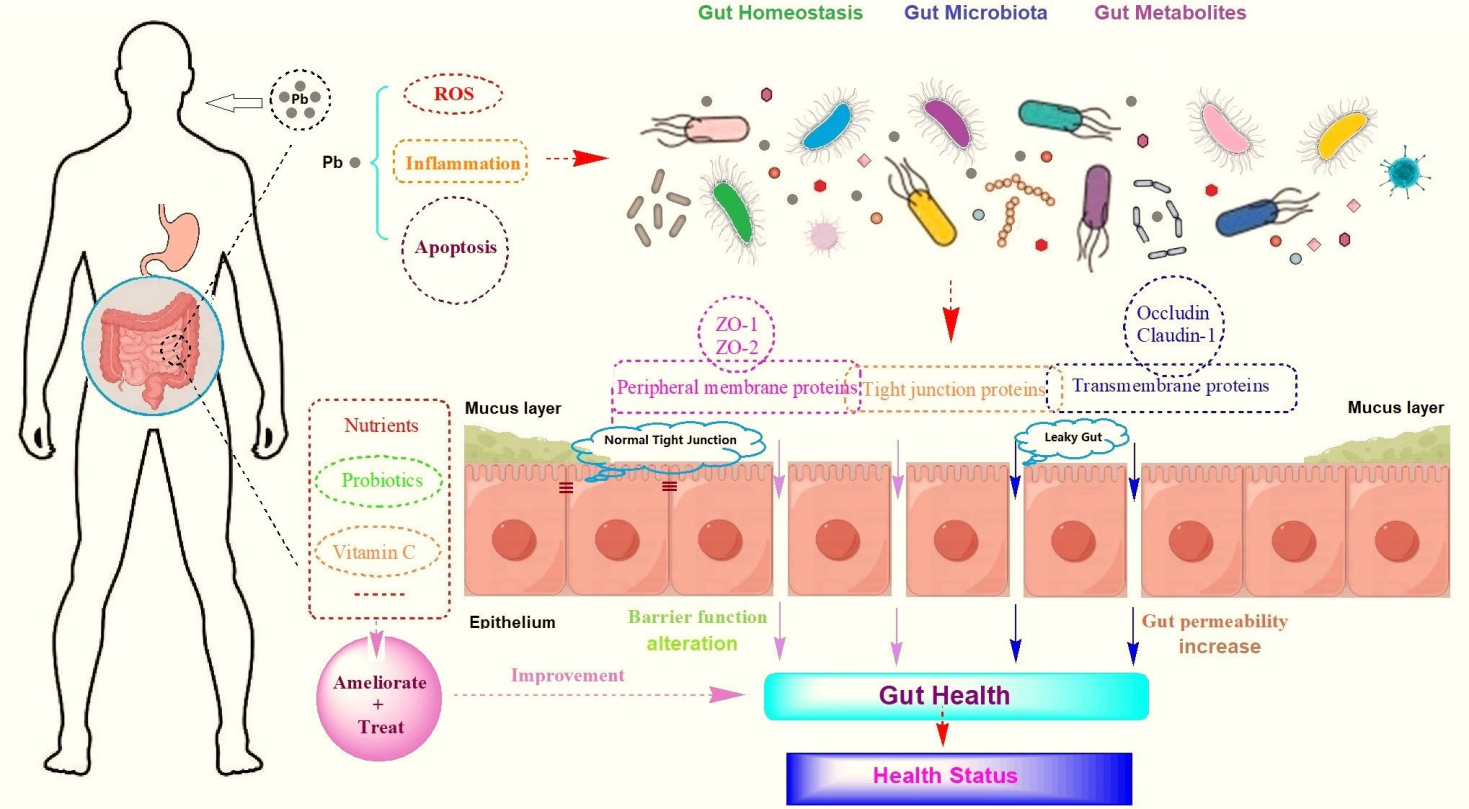
Impact of Pb toxicity on gut homeostasis, microbiota, and metabolites. Pb can increase gut permeability and alter barrier function to induce ROS, inflammation, and apoptosis, which further influence gut health and health status of the body, and vice versa.

The estimated daily average Pb intake among consumers varies significantly across countries: Germany (37.1 µg/day), China (35.1 µg/day), Spain (14.7 µg/day), Korea (9.8 µg/day), and the United States (3.5 µg/day) (39). Notably, these values exceed the US Food and Drug Administration’s (FDA) provisional tolerable intake limit of 12.5 µg/day, indicating potential health risks from excessive dietary Pb exposure in Germany, China, and Spain (40, 41). Consistent with these findings, mean blood Pb levels in children also reflect regional disparities, with concentrations of 3.71 µg/dL in China, 1.10 µg/dL in Germany, and 0.84 µg/dL in the United States (18–20). It is worth noting that Pb exposure accounts for 0.6% of global disease burden, 21.7 million disability-adjusted life years, and nearly 1 million annual deaths (9, 42). Children and LMICs bear the brunt of this silent epidemic, necessitating stricter industrial regulations, enhanced waste management, and targeted public health interventions. Although exposure to Pb contributes to the disturbance of inflammatory cytokines levels in children and the alteration of the gut system, its effects on gut homeostasis, microbiota, and metabolites are still unclear (Fig. 1) (37). Future research should elucidate Pb-gut microbiome interactions to develop novel therapeutics (e.g., probiotics, metal-chelating agents) for vulnerable populations.

## REVIEW METHOD

To ensure a comprehensive and systematic literature search, the following electronic databases were queried up to May 2025: Web of Science, PubMed, Science Direct, SpringerLink, Wiley Online Library, American Chemical Society (ACS) Publications, and Google Scholar. The search terms were strategically grouped into five key conceptual categories: Lead/Heavy Metals: (“lead” OR “Pb” OR “heavy metal”); Gut/Intestinal System: (“gut” OR “intestinal” OR “intestine”); Homeostasis: (“homeostasis” OR “balance” OR “equilibrium”); Microbiota: (“microbiota” OR “flora” OR “microbiome” OR “bacteria”); Metabolites/Metabolism: (“metabolite” OR “metabolize” OR “metabolism” OR “metabolome”). Broad search terms were employed to maximize the retrieval of relevant publications while minimizing exclusion bias. All identified studies were independently evaluated by two authors to ensure consistency and reliability in the selection process. The search was restricted to English-language publications to maintain linguistic uniformity in the analysis.

## Pb TOXICITY ON GUT HOMEOSTASIS

**Fig 2:**
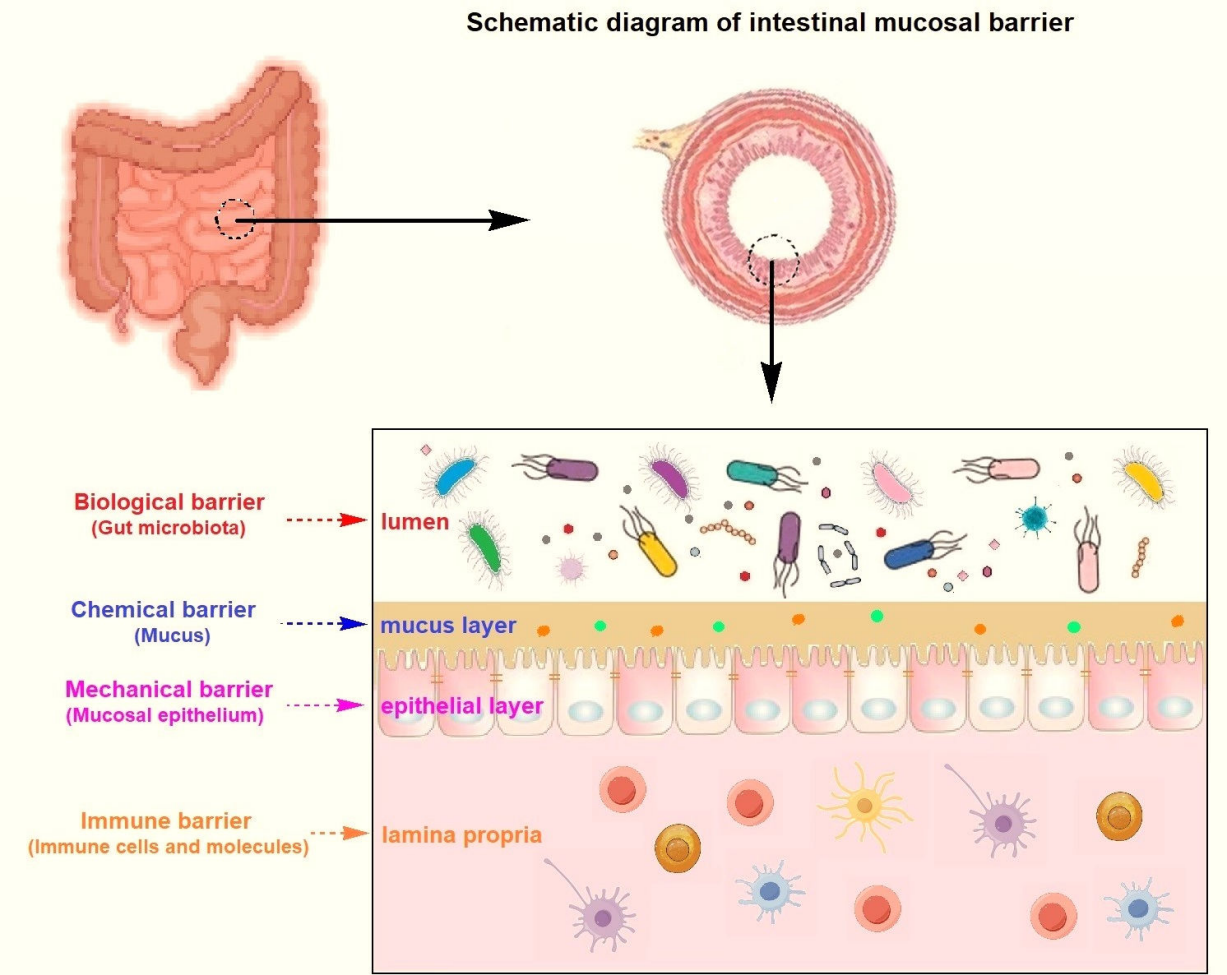
Schematic diagram of intestinal mucosal barrier. It can be roughly divided into four categories: biological barrier (gut microbiota), chemical barrier (mucus), mechanical barrier (mucosal epithelium), and immune barrier (immune cells and molecules).

The structure and function of the intestinal mucosal barrier are crucial for maintaining gut homeostasis (Fig. 2). While extensive research has emphasized establishing a healthy gut microbiota through optimized dietary patterns and balanced nutrient intake to support beneficial bacteria, emerging evidence reveals that lead (Pb) exposure can significantly compromise this delicate equilibrium. Pb exposure induces direct and indirect alterations in gut microbiota composition, disrupting intestinal homeostasis and ultimately impairing host health (43). A compelling cross-sectional study involving preschool children demonstrated striking differences between Pb-exposed and reference groups. The exposed children exhibited elevated blood Pb levels, increased plasma bacterial endotoxins, higher incidence of diarrhea, and impaired physical development compared to their unexposed peers. Notably, a strong positive correlation emerged between blood Pb concentrations and plasma endotoxin levels, suggesting Pb exposure leads to enhanced intestinal permeability and gut dysfunction (44). Known as lipopolysaccharide (LPS), bacterial endotoxin is currently considered a novel and practical bacterial biomarker, which is shed from the outer membranes of dead Gram-negative bacteria. It can be transported from the gut to the circulation through an incomplete intestinal mucosal barrier. The bacterial endotoxin, lipopolysaccharide (LPS), has gained recognition as a clinically relevant microbial biomarker due to potent pathogen-associated molecular pattern. This Gram-negative bacterial membrane component translocates into systemic circulation when intestinal barrier integrity is compromised (45–47). It activates host immune responses by binding to Toll-like receptors, which triggers MyD88 and TRIF signaling pathways, culminating in the production of pro-inflammatory cytokines including interleukin-1β, interleukin-6, and tumor necrosis factor-α (48–50).

Animal studies have manifested that both short- and long-term Pb exposure increases gut leakage, while the loss and alteration of gut microbiota exacerbate gut barrier damage in Pb-exposed mice (Table 1). For example, a previous study reported that antibiotics were first used to deplete the intestinal microbial community in mice, and then the mice were acutely exposed to Pb by gavage with a dose of 1,304 mg/kg lead acetate (PbAc) for 3 days. The results showed that elevated serum concentrations of DX-4000-FITC (fluorescein isothiocyanate-4000), reduced expression of peripheral membrane proteins [Zona Occludens 1 (*ZO-1*) and 2 (*ZO-2*)], and transmembrane proteins (Claudin-1 and Occludin) were found in the colon and jejunum of acutely Pb-exposed mice. After 8 weeks of exposure to natural drinking water containing 1.83 g/L PbAc, the expression of tight junction proteins (TJPs) in the colon and jejunum tissues of mice significantly decreased compared to the control group (31). TJPs, including transmembrane proteins and peripheral membrane proteins, are crucial for cell adhesion, binding gaps between adjacently polarized epithelial cells or endothelial cells and maintaining barrier function (51).

**TABLE 1:** Effects of lead (Pb) exposure on gut health based on animal studies

Effect or outcome	Reference
Exposure to Pb decreases the expression of tight junction protein in colon and jejunum tissues in mice	(31)
Significant inflammatory cell infiltration and edema were found in lead-exposed mice	(32)
Low concentrations of exposure to Pb induce metabolic disorder and gut microbiota dysbiosis in mice	(33)
Chronic lead exposure exacerbated hepatic glucolipid, metabolism disorder, and gut microbiota dysbiosis in high-fat diet mice	(35)
Lead exposure caused tissue damage, inflammation, downregulated expression of occluding protein and ZO-1 mRNAs	(51)
Emblica officinalis alleviated gut toxicity of mice by improving gut microbiota with Pb exposure	(52)
Lead exposure induced structural damage, digestive stress, immune response, and microbiota dysbiosis in the intestine of silver carp	(53)
Carp with lead exposure showed increased LPS and intestinal tissue injury, decreased ZO-1, occluding expression, and gut diversity	(54)
Long-term Pb exposure induced histopathological damage and microbiota dysbiosis in the cecum of female Japanese quails	(55)
Pb exposure significantly alters gut microbiota composition and results in weight gain of adult mice	(56)
Lactobacillus and Bifidobacterium were significantly lower in the Pb-exposed flies when compared with reference	(57)
Exposure to Pb-halide perovskite nanoparticles could deliver bioavailable Pb without altering endogenous gut microbiota in zebrafish	(58)
Short-time exposure to Pb could cause gut microbiota dysbiosis and hepatic metabolic disorder in adult male zebrafish	(59)
Exposure to Pb affected gut microbial richness (alpha diversity)	(60)
Long-term lead exposure induced fatty liver disease and related alterations of gut microbiota in rats	(61)
Gut microbiota played a mediating role in the links between prenatal maternal lead exposure and neurodevelopmental deficits in rats	(62)
Pb exposure aggravated glucose metabolism disorders in high-fat diet-fed mice	(63)
Pb exposure exacerbated liver injury by disrupting gut microbiota and related metabolites in high-fat diet-fed mice	(64)

Moreover, Pb exposure induced colon tissue inflammation and lesions in C57BL/6 mice, evidenced by downregulated expression of Occludin protein and *ZO-1* mRNA in colonic epithelial cells (32). Notably, treatment with *Emblica officinalis* (*E. officinalis*) fruit extract alleviated Pb-induced damage, improving weight loss, shortening colon length, and increasing TJP expression (Claudin-1, Occludin, and *ZO-1*) in a dose-dependent manner (52). It also reduced inflammatory cell infiltration in colonic tissue.

Interestingly, in silver carp, 96 hours of Pb exposure severely damaged the gut barrier, leading to intestinal leukocyte infiltration, increased goblet cells, shortened intestinal villi, thickened gut wall, downregulated structural genes (villin-1 and claudin-12), and upregulated expression of pro-inflammatory factors (IL-8 and TNF-α). These findings suggest that Pb promotes intestinal inflammation and disrupts intestinal structure (53).

Similarly, common carp exposed to Pb-exhibited elevated serum LPS, intestinal tissue injury, decreased ZO-1 and occludin expression, and reduced gut bacterial diversity (54). In addition, chronic Pb exposure also caused pathological damage to the cecal tissue of female Japanese quails (*Coturnix japonica*), characterized by exfoliation of mucosa, destruction of Lieberkühn glands, and proliferation of lymphocyte. Ultrastructural damage included nuclear condensation, mitochondrial vacuolization, and microvillus contraction. Specially, Pb exposure altered immune-related gene expression in the ceca, downregulating interleukin-2 (*IL-2*) and interferon gamma (*IFN-γ*), whereas upregulating interleukin-6 (*IL-6*), tumor necrosis factor-alpha (*TNF-α*), and nuclear factor-kappa B (*NF-κB*) (55).

An in *vitro* study further confirmed that Pb exposure induced gut damage in a dose-dependent manner. Human colon cancer HT-29 cells exposed to 8 mM PbAc for 24 hours showed reduced viability, increased intracellular ROS levels, and suppressed expression of TJPs (*ZO-1* and *Occludin*) (32). In summary, the severity of Pb-induced gut damage depends on exposure concentration, with mechanisms involving tissue structural changes, immune dysfunction (oxidative stress and inflammatory substances), and microbiota dysbiosis.

In summary, Pb may influence TJPs via the following pathways. First, Pb exposure significantly reduces the mRNA and protein expression of key tight junction (TJ) components, including ZO-1, occludin, and claudins, through multiple mechanisms: (i) PKC and MAPK/ERK Activation: Pb triggers Protein Kinase C (PKC) and MAPK/ERK signaling, leading to the phosphorylation-dependent suppression of occludin and other TJ proteins. (ii) Epigenetic Silencing: Pb induces DNA hypermethylation and histone deacetylation at the promoters of TJ genes (e.g., ZO-1, occludin), effectively silencing their transcription. (iii) Oxidative Stress & Redox Imbalance: Pb generates reactive oxygen species (ROS), which further destabilize TJ complexes by altering their structural integrity. Second, Pb-induced gut barrier integrity and dysfunction is closely linked to pro-inflammatory signaling cascades: (i) NF-κB Activation: ROS generated by Pb exposure activates NF-κB, a master regulator of inflammation, which suppresses TJ protein expression and promotes cytokine release (e.g., TNF-α, IL-6). (ii) Pb stimulates MAPK (p38, JNK, ERK) signaling, leading to AP-1 activation, which further represses TJ genes and exacerbates intestinal permeability. (iii) Nrf2 suppression: Pb disrupts the Nrf2 antioxidant pathway, impairing cellular defenses against oxidative damage and perpetuating TJ dysfunction (34, 65–69).

## Pb TOXICITY ON GUT MICROBIOTA AND MICROBIOME

Gut microbiota plays a crucial role in maintaining barrier structure and function, as well as influencing the development of a variety of diseases, including cardiovascular, immune, neurological, skeletal, and gastrointestinal (GI) disorders (70–72). Previous studies demonstrated that exposure to Pb can cause multisystem damage, but its specific impact on the GI system and linked gut microbiota warrants further investigation (73, 74). Different gut bacteria respond to Pb exposure in varying ways, sometimes with delayed effects. Pb primarily alters gut microbiota by reducing microbial diversity and shifting community composition. Even without eliminating specific species, Pb can disrupt microbial structure, with longer or higher exposures leading to more pronounced changes.

The two most abundant phyla in the human gut microbiota are *Firmicutes* and *Bacteroidetes*, and their ratio (F/B ratio) serves as a potential notable biomarker reflecting the health status of the body (75–77). As the largest gram-negative phylum in the GI tract, Bacteroidetes is the cornerstone of maintaining a healthy balance in the GI tract. It helps maintain gut balance, aids in nutrient digestion and calorie absorption, and regulates immune function. These bacteria degrade polysaccharides through specialized enzymes, fostering a symbiotic relationship with the host and supporting immune system maturation. Bacteriophages are generally considered to be beneficial to the human body under normal abundance (78–80). Additionally, *Firmicutes* are major producers of butyric acid, which converts to butyrate—a beneficial fatty acid critical for GI health and metabolic regulation. Reduced abundance of butyrogenic *Firmicutes* has been linked to type 2 diabetes (81–83).

Notably, an elevated F/B ratio is associated with obesity and may contribute to gallstone formation (84). Pb exposure tends to increase *Firmicutes* while decreasing *Bacteroidetes*, disrupting the F/B ratio and potentially harming health (56, 85–87). Other important microbial ratios include B/E ratio (the ratio of *Bifidobacteria* to *Enterobacteriaceae/Escherichia coli*) is used to estimate the extent of gut microecology dysbiosis and microbial colonization resistance (88–90). Moreover, the C/B ratio (the ratio of *coccus*/*bacillus*) reflects gut bacterial homeostasis (91). Furthermore, the K/B ratio (the ratio of *Klebsiella*/*Bifidobacterium*) is an underlying indicator/marker for early infant allergies (92).

In Drosophila, Pb exposure reduced Lactobacillus and Bifidobacterium, correlating with impaired memory and social behavior (57). In addition, Pb-contaminated food altered gut microbiota composition, increasing Pseudomonas abundance and triggering hepatic metabolic disorders in zebrafish (58, 59). Pb exposure dose-dependently increased *Marvinbryantia* and *Ruminococcus 1* while decreasing *Lactobacillus* and *Roseburia*. Chronic exposure also induced fatty liver disease and gut dysbiosis (34, 60). Long-term Pb exposure induces fatty liver disease and related alterations of gut microbiota in rats (61). Maternal Pb exposure in rats disrupted offspring neurodevelopment via gut microbiota mediation (62). Furthermore, Pb exposure aggravates liver injury and glucose metabolism disorders by damaging gut microbiota, metabolites, and barrier in high-fat diet-fed mice (63, 64). These results suggest that chronic Pb exposure disrupts trace element balance and induces dose-dependent gut dysbiosis, differing from acute Pb poisoning effects (34).

Population-based studies on the impact of Pb exposure to gut microbiota not only are extremely limited but also show inconsistent results (Table 2). A US cross-sectional study linked urinary Pb levels to increased microbial α-diversity and richness and higher phylum *Proteobacteria* and the order *Burkholderiales* (93). The abundance levels of *Bifidobacterium*, *Lactobacillus*, and *Escherichia coli* were significantly lower in children with high blood Pb levels than those in the control group, and the B/E ratio <1 indicates that the structure of the gut microbiota has been disrupted and the resistance of the gut has decreased due to Pb exposure (94). A randomized trial reported that elevated blood Pb was linked with ascending relative abundance levels of fecal *Gammaproteobacteria* and *Succinivibrionaceae* (95). A cohort study found maternal Pb exposure correlated with higher *Malassezia* and *Saccharomyces* but lower *Candida* and *Aspergillus* in children. Prenatal/postnatal Pb exposure altered infant microbiota, increasing Collinsella and Bilophila while reducing Bacteroides (96, 97). Prenatal Pb exposure was negatively associated with childhood gut microbiota diversity and F/B ratio (98). Second-trimester Pb exposure was positively associated with a two-taxa microbial clique [OR (CI), 1.03 (1.01 to 1.05)] below the median relative abundance of *Bifidobacterium adolescentis* and *Ruminococcus callidus* in children of 9–11 years old (99). Our recent cross-sectional study from an e-waste dismantling area found that both blood and urinary Pb levels were positively associated with α-diversity, while negatively associated with β-diversity; a variety of significant differential species were found in children between the high-Pb-exposed group and the low-Pb-exposed group (100). Taking together, the differences of Pb in relation to gut microbiota between the studies may be due to various study design, demographic characteristics of the participants, sample size and type, exposure time and dose, and test methods.

**TABLE 2:** Effects of lead (Pb) exposure on gut health of population-based studies

Effect or outcome	Reference
Elevated peripheral blood endotoxin and inflammatory indices linked with increased intestinal permeability in children with e-waste lead exposure	(44)
Urinary lead concentrations in relation to composition of gut microbiota such as α-diversity and β-diversity	(93)
High blood lead changed the composition of gut microbiota in children	(94)
Elevated blood lead was associated with increases in Succinivibrionaceae and Gammaproteobacteria relative abundance levels in the stool of school-aged children	(95)
Fetal and early postnatal lead exposure measured in teeth associated with infant gut microbiota	(96)
Significantly negative associations were found between childhood blood Pb and acetylene degradation pathway abundance	(97)
Prenatal Pb exposure was inversely associated with childhood gut microbiome	(98)
Prenatal Pb exposure was related with declined abundance of beneficial gut microbial cliques in childhood	(99)
E-waste Pb exposure led to significant change of gut microbiota and metabolomics in children	(100)
SYF-08 was identified as Lacticaseibacillus casei exhibiting a Pb^2+^-binding capacity and Pb^2+^ tolerance, which increased gut microbiota diversity of the offspring	(101)

Probiotics have emerged as a prominent area of research for enhancing gut health and managing various diseases in recent years (102, 103). Of which, Actinobacteria, although only accounting for a small proportion of the gut microbiota, plays an important role in maintaining gut homeostasis. Several probiotic species within this phylum contribute to the establishment of a healthy gastrointestinal defense barrier and support metabolic processes (104). Notably, a decline in *Bifidobacterium*, a key Actinobacteria probiotic, has been linked to immune system activation and the onset of negative emotional states such as anxiety and depression (105). These findings underscore the importance of Actinobacteria in preserving gut homeostasis and supporting overall physiological function.

Interestingly, probiotics have been found to have the potential to mitigate or reverse gut damage caused by Pb exposure in recent years. For instance, *Lactobacillus plantarum CCFM8661* has been shown to bind Pb *in vitro,* reducing its levels in blood and tissues while preventing ROS by modulating antioxidant enzymes like glutathione, glutathione peroxidase, malondialdehyde, superoxide dismutase in mice (106, 107). Additionally, gut microbes such as *Escherichia coli* and *Lactobacillus acidophilus* can decrease intestinal Pb absorption, thereby mitigating its toxicity. This protective effect may result from either direct reinforcement of the gut barrier or indirect sequestration of Pb through bacterial adsorption (108).

Oral administration of Pb-intolerant probiotics, including *Faecalibacterium prausnitzii* and *Oscillibacter ruminantium,* has been found to restore gut barrier integrity by upregulating tight junction proteins (*ZO-1*, *ZO-2*, *Occludin*, and *Claudin-1*) in the colon and small intestine. These probiotics also reverse Pb-induced reductions in short-chain fatty acids (SCFAs), such as acetic acid and butyric acid, produced by colonic bacteria (31). Similarly, supplementation with *Bacteroides thetaiotaomicron FTJS-8-K* enhances fecal Pb excretion, reduces tissue Pb accumulation, and alleviates oxidative stress. It also upregulates *ZO-1* and *Occludin* mRNA expression, improving gut permeability and promoting SCFA production (109). Certain dietary ingredients, such as oxalates, phosphates, and phytates, further reduce Pb solubility and absorption, minimizing its toxic effects (101).

In a screening study to identify Pb-mitigating probiotics from infant gut microbiota, SYF-08, isolated from infant stool samples, exhibited promising protective properties against Pb toxicity (110). Beyond probiotics, ascorbic acid (vitamin C) has been widely studied for its antioxidant and chelating properties, making it an effective adjunct in Pb poisoning treatment by maintaining redox balance (111). Moreover, dietary supplementation with fiber and galactooligosaccharide (GOS) has been shown to enhance fecal Pb excretion, reduce blood and tissue Pb levels, and restore gut microbiota composition, barrier function, bile acid metabolism, and essential nutrient utilization in mice (112, 113).

In summary, probiotics can enhance gut barrier by upregulating TJPs, reducing oxidative stress, and modulating metabolic byproducts such as SCFAs induced by Pb. Pb exposure disrupts the gut microbiota composition, leading to reduced levels of beneficial SCFAs such as acetate, propionate, and butyrate through potential mechanisms. ① Depletion of SCFA-Producing Bacteria: Pb selectively inhibits commensal microbes (e.g., Lactobacillus, Bifidobacterium, Faecalibacterium prausnitzii) that ferment dietary fiber into SCFAs, and probiotic supplementation (e.g., Pb-tolerant Lactobacillus strains) can restore SCFA levels and mitigate Pb toxicity by competitive exclusion of Pb-absorbing pathogens (31). ② Impaired Microbial Metabolism: Pb directly interferes with bacterial enzymatic pathways (e.g., butyryl-CoA transferase) critical for SCFA synthesis, and SCFAs readily regulate immune cells (e.g., Tregs) and alleviate inflammation via activating GPR41/43 (63, 64). These findings highlight the microbiota-SCFA-host axis as a critical mediator of Pb’s metabolic toxicity and a potential target for dietary or microbial therapies.

Probiotics, dietary fiber, and vitamin C in mitigating Pb toxicity mainly involve in the following processes. First, probiotics (e.g., *Lactobacillus*, *Bifidobacterium*) compete with Pb-absorbing pathogens for intestinal binding sites, reducing Pb bioavailability (114). In addition, bacterial cell wall components (teichoic acids, peptidoglycans) directly chelate Pb ions and microbial metabolites (e.g., phytochelatins) facilitate Pb sequestration (114, 115). Moreover, dietary fiber may restore gut microbiota balance and alleviate Pb- induced neurotoxicity through generating SCFA in the presence of beneficial intestinal bacteria such as *Lactobacillus* and *Bifidobacterium* that secrete cellulases and hemicellulases (113, 116). Some of SCFA as butyric acid is not only the main energy source for colonic epithelial cells maintaining the integrity of the intestinal barrier and reduce inflammation but also promoting TJP expression to reduce intestinal leakage. Different types of dietary fiber provide substrates for unique bacteria, promoting microbial diversity and gut health. Notably, insoluble fiber increases stool bulk and accelerates intestinal motility, thereby reducing the retention time of harmful substances in the gut. Vitamin C, as an antioxidant, neutralizes Pb-induced ROS through redox cycling of ascorbate/dehydroascorbate, restores activity of Pb-inhibited antioxidant enzymes (SOD, CAT, GPx), and activates the Nrf2/ARE pathway to boost endogenous antioxidants (117–119).

Most supporting evidence comes from animal models. Few human clinical trials exist for vitamin C intervention. A study from India found that vitamin C supplementation does not effectively lower blood lead levels in battery manufacturing workers, it demonstrates significant antioxidant benefits by reducing lipid peroxidation and nitrite formation. Furthermore, it enhances antioxidant enzyme activity in erythrocytes, particularly superoxide dismutase and catalase (120). These mechanisms collectively limit Pb absorption, promote its excretion, and mitigate Pb-induced gut damage.

## Pb TOXICITY ON GUT METABOLITES

Gut microbes communicate with the host through metabolites which are small biomolecules produced as intermediate or end products of microbial metabolism. A balanced gut microbiota plays a beneficial role in nutrient digestion, particularly by breaking down dietary fiber to enhance nutrient absorption and maintain overall health. However, lead (Pb) exposure not only disrupts gut structural integrity and microbial balance but also induces metabolic dysfunction, further compromising gut health (121).

Chronic Pb exposure (0.1 mg/L Pb for 15 weeks) in mice significantly altered relative abundance of *Firmicutes* and *Bacteroidetes* and modified 15 key metabolites, disrupting critical metabolic pathways such as amino acid metabolism and tricarboxylic acid (TCA) cycle (33). Multi-omics analyses reveal that Pb exposure broadly influences gut metabolites such as *glucose*, *vitamin E*, *bile acids*, while also impairing metabolic pathways such as nitrogen, energy, and lipid metabolisms. Notably, Pb reduces the production of SCFAs such as acetic, propionic, butyric, isobutyric, and valeric acids in colon contents and feces (31, 109). These disruptions destabilize the gut environment, impairing SCFA secretion, lipid metabolism, and bile acid metabolism, ultimately weakening the intestinal epithelial layer (122).

Our previous epidemiological study in children further demonstrated that a total of 19 differential metabolites were significantly identified between the high Pb exposed group and the low Pb exposed group. Of which, 13 metabolites were upregulated and 6 metabolites were downregulated. These changes were linked to critical pathways, including sphingolipid signaling, amino acid metabolism, tRNA biosynthesis, bile secretion, and cell death mechanisms (apoptosis and necroptosis) (100). It is noticeable that the severity of these effects depends on exposure dose and potential interactions with other environmental contaminants, highlighting the need for further research on combined exposures (123).

## CONCLUSION

Lead (Pb) exposure can significantly compromise gut barrier function by directly inducing histopathological changes and immune dysregulation, including elevated oxidative stress and inflammatory mediators. Additionally, Pb indirectly disrupts gut homeostasis by altering the gut microbiota’s diversity, composition, and metabolic activity, further exacerbating intestinal permeability and systemic toxicity. Notably, Pb influences both microbial-derived and host-derived metabolites, modulating critical metabolic pathways that contribute to disease progression.

Current evidence highlights that certain nutrients such as probiotics and vitamin C may serve as effective detoxifying agents by enhancing Pb excretion, reducing absorption and accumulation, and mitigating its toxic effects. Probiotic supplementation, in particular, represents a promising strategy for preventing and alleviating Pb poisoning. However, while preclinical studies in animal models demonstrate its efficacy, further research is needed to validate these findings in human populations, especially in Pb-contaminated areas, and to elucidate the precise mechanisms underlying probiotic-mediated protection.

To drive progress in this field, several critical research priorities demand focused exploration. (i) Multi-omics integration—combining genomics, proteomics, metabolomics, and microbiomics could unravel the intricate interplay between Pb exposure, gut microbiota shifts, and host metabolic disruptions. (ii) Personalized interventions—exploring gut microbiota profiling to tailor targeted therapies for individuals with high Pb exposure. (iii) Dietary modifications—investigating the role of prebiotics, fiber, and other dietary components in reinforcing gut resilience against Pb-induced damage. (iv) Translational studies—bridging the gap between animal models and human trials to optimize probiotic strains, dosages, and treatment durations for clinical applications. In summary, addressing these gaps will not only deepen our understanding of Pb toxicity but also pave the way for practical, microbiota-based interventions to safeguard vulnerable populations.

## Data Availability

No new data were created or analyzed in this study. Data sharing is not applicable to this article.
